# IL23-axis cytokines and C-reactive protein as dissociable biomarkers of vascular recovery in psoriasis: A hypothesis

**DOI:** 10.1007/s12026-026-09876-x

**Published:** 2026-11-09

**Authors:** Yanbing Zhou

**Affiliations:** Department of Kinesiology and Health Education, The University of Texas at Austin, 2109 San Jacinto Blvd, D3700, Austin, TX 78712 USA

**Keywords:** Systemic inflammation, Vascular dysfunction, Cardiovascular risk, Arterial stiffness

## Abstract

Biologic therapy has transformed psoriasis into a clinically controllable disease, yet skin clearance does not guarantee resolution of the systemic inflammation and vascular dysfunction that drive cardiovascular risk in this population. We hypothesize that interleukin (IL)-23-axis-proximal cytokines and C-reactive protein (CRP) mark two distinct, dissociable inflammatory compartments in psoriasis: IL23-axis-proximal signaling predominantly governs cutaneous disease activity and microvascular endothelial function, whereas CRP integrates a broader cardiometabolic burden and tracks macrovascular recovery. We distinguish IL23-axis-proximal cytokines (IL23, IL17a, IL22), whose production is largely confined to lesional skin, from shared effector cytokines (TNF-α, IFN-γ, IL1, and IL6), which also arise from adipose tissue and other systemic sources. This model generates two falsifiable predictions: (1) IL23 blockade will improve cutaneous microvascular endothelial function in proportion to suppression of IL23-axis-proximal signaling and out of proportion to any change in CRP, and (2) persistent CRP elevation despite adequate skin clearance will identify patients with blunted macrovascular recovery even when IL23-axis-proximal signaling is suppressed. Because IL6 is a node shared by cutaneous and adipose inflammation, the two compartments are coupled, and we therefore state the model in relative rather than absolute terms: quantitative dissociation, not statistical independence. If confirmed, this compartmentalized model would explain why near-complete skin clearance under biologic therapy, including IL23 blockade, does not uniformly normalize cardiovascular risk, and would offer two complementary, mechanism-specific biomarkers, rather than skin clearance alone, for monitoring vascular recovery after psoriasis treatment. We outline the rationale for this hypothesis and a longitudinal biomarker-imaging design capable of refuting it.

## Introduction and hypothesis

Psoriasis confers substantially elevated cardiovascular risk, with excess prevalence of dyslipidemia, diabetes, hypertension, obesity, low high-density lipoprotein (HDL) cholesterol, and metabolic syndrome [1, 2], and increased cardiovascular mortality relative to the general population, particularly with greater disease severity [3]. Biologic therapy, especially interleukin (IL)-23 blockade, now achieves near-complete skin clearance in most patients [4], yet whether this cutaneous response reflects parallel resolution of the systemic inflammation and vascular dysfunction that drive cardiovascular risk remains unresolved [5, 6]. This uncertainty leaves clinicians without a validated way to determine, from skin status alone, whether a treated patient’s cardiovascular risk has actually declined.

We hypothesize that IL23-axis-proximal cytokines and C-reactive protein (CRP) mark two distinct, dissociable inflammatory compartments in psoriasis. IL23-axis-proximal signaling, defined here as IL23 itself together with the cytokines whose production is largely restricted to the IL23/Th17-activated lesion (IL17a, IL22), predominantly governs cutaneous disease activity and microvascular endothelial function, the vascular bed embedded within and adjacent to psoriatic skin. We deliberately separate these axis-proximal cytokines from shared effectors (TNF-α, IFN-γ, IL1, and IL6), which are amplified by IL23/Th17 activation but are also produced by adipose tissue, macrophages, and circulating leukocytes independently of cutaneous disease. CRP, by contrast, integrates a broader, multi-sourced cardiometabolic signal, hepatic, adipose, and cytokine-derived, and tracks macrovascular structure and function, including arterial stiffness and conduit-artery endothelial dilation. The two compartments are coupled rather than isolated: IL6 is induced both by lesional keratinocyte and immune activation and by adipose tissue, and is the dominant hepatic inducer of CRP. Our claim is therefore quantitative. If the compartments are dissociable in the sense we propose, IL23 blockade should improve microvascular function in proportion to axis-proximal suppression and out of proportion to the change in CRP, while persistent CRP elevation despite adequate skin clearance should flag patients with blunted macrovascular recovery even when IL23-axis-proximal signaling is suppressed. This distinction, if confirmed, would explain a pattern already visible in the literature: near-complete skin clearance under biologic therapy, including IL23 blockade, without full normalization of residual inflammation or vascular function [5–7], and would support supplementing skin clearance with two complementary, mechanism-specific biomarkers for monitoring vascular recovery after treatment.

## Two inflammatory pathways with distinct drivers

The mechanistic case for compartmentalization begins with the biology of each axis. The IL23/Th17 immune axis is the dominant driver of psoriatic pathogenesis and is independently implicated in microvascular endothelial dysfunction [8], while TNF-α/IFN-γ synergism is more specifically linked to psoriatic atherogenesis [9]. TNF-α and IFN-γ are induced within psoriatic lesions but are not axis-restricted, being produced also by adipose-tissue macrophages and circulating myeloid cells; we therefore treat them throughout as shared effectors rather than as IL23-axis-proximal cytokines. Cytotoxic CD8 + resident memory T cells, macrophages, and natural killer T cells provide additional cytokine sources within lesional skin [10, 11]. Cytokine-activated keratinocytes upregulate adhesion molecules and vascular endothelial growth factor, promoting leukocyte trafficking and increased vascular permeability [12], while cytokine-driven inducible nitric oxide synthase (iNOS) activity generates nitric oxide (NO), at concentrations that remain debated, which favors keratinocyte hyperproliferation over vasodilation [13, 14], a self-amplifying cycle that sustains the cutaneous inflammatory “storm” characteristic of active plaques [15, 16].

This lesional, IL23/Th17-driven signature extends into the cutaneous microcirculation. Oxidative stress degrades NO before it can relax vascular smooth muscle, producing measurable cutaneous microvascular endothelial dysfunction and abnormal microvascular conductance in active disease [17–19]. iNOS expression has been localized to the papillary dermis, which contains the dilated, tortuous capillary loops characteristic of psoriatic plaques, where it correlates with leukocyte infiltration, while eNOS expression remains comparable to healthy skin [20, 21]. The nitric oxide literature on lesional skin is not fully consistent, and the discrepancy is worth stating explicitly. Earlier tissue-level studies reported markedly increased iNOS expression and nitrite output in lesional skin, with NO production reported at up to 200-fold that of healthy skin [22]. By contrast, Köhler et al. found strong, IL17a-dependent NOS2 mRNA expression in psoriatic epidermis; in cultured keratinocytes, IL17a was required for NOS2 induction, which was significant in combination with TNF-α and greatest with the further addition of IL22, whereas NOS2 protein was only marginally increased, an effect they attribute to IL17a-induced miR-31, probably acting indirectly. In the same study, low rather than high NO concentrations increased keratinocyte proliferation and mediated IL17a downstream gene expression [23]. These findings have not yet been reconciled. Köhler et al. also found no increase in nitrotyrosine in psoriatic skin and identified the epidermis as the main source of NOS2 mRNA [23], and citrulline, an end product of NOS2 activity, has been reported to be equally present in psoriatic and control skin [24], whereas earlier work localized iNOS mainly to the papillary dermis [20, 21]. Differences in cellular source (epidermal keratinocytes versus dermal immune cells), in what was measured (protein or enzyme products versus nitrite), and in disease stage may account for the discrepancy, but this explanation remains speculative. The implication for the present hypothesis is the same under either reading: NO in lesional skin is iNOS-derived and immunologically driven rather than eNOS-derived and vasodilatory, and both its transcriptional induction, which requires IL17a and is amplified by TNF-α, and its post-transcriptional restraint by IL17a-induced miR-31 are gated by IL17a. This is therefore a mechanistically local, IL23-axis-proximal phenomenon, and the argument does not depend on the absolute magnitude of lesional NO.

CRP arises through a different, less anatomically constrained route. Hyperproliferative keratinocytes and activated immune cells release IL6, which travels to the liver and triggers CRP synthesis [25]. Circulating CRP is elevated in psoriasis and tracks skin disease activity independent of BMI, age, and sex [26], and vascular inflammation increases with psoriasis duration, contributing roughly a 1% increase in cardiovascular risk per additional year of disease [27], with reductions in skin activity linked to decreased aortic vascular inflammation [28]. Critically, however, IL6, which also arises substantially from adipose tissue independent of skin disease, is the dominant hepatic inducer of CRP [29]. The quantitative weight of this non-cutaneous input is central to the hypothesis, and it can be bounded with existing data. Arteriovenous sampling in severely obese humans (mean BMI ≈ 55 kg/m²) shows portal-vein IL6 concentrations approximately 50% higher than radial-artery concentrations, with portal IL6 correlating directly with systemic CRP (*r* = 0.54) [30], and sampling across a subcutaneous abdominal depot shows that adipose tissue releases IL6 into the systemic circulation in vivo, in amounts extrapolated to account for an estimated 15–25% of systemic IL6 around midday and 25–35% in the evening [31]. The cutaneous contribution appears modest at the individual-patient level: across 186 treatment cycles with TNF-α and IL12/23 antagonists, PASI correlated only weakly to moderately with CRP (median within-patient Spearman ρ = 0.20), with wide inter-individual spread (interquartile range 0.6), and even complete skin clearance (PASI 0) identified only 24% of cycles with CRP below the cardiovascular-risk threshold of 3 mg/L [32]. A median correlation of this size implies that, in a typical patient, skin activity and circulating CRP share only about 4% of their rank variance (ρ² ≈ 0.04), although the wide spread indicates a larger cutaneous contribution in a minority of patients. This estimate comes from patients treated with TNF-α and IL12/23 antagonists rather than IL23-selective agents, CRP was measured with a conventional assay left-censored at 2 mg/L, which may attenuate correlations at low concentrations, and PASI is an imperfect proxy for cutaneous cytokine output, so it should be read as an order-of-magnitude bound to be tested directly in the proposed design. CRP is best described as a multi-sourced cardiometabolic integrator with a minority cutaneous contribution, rather than as a direct downstream marker of IL23-axis signaling. Accordingly, we do not claim that the compartments are independent; we claim that the shared IL6 node is small enough, relative to adipose and hepatic inputs, for microvascular and macrovascular recovery to separate detectably along the two biomarkers. Stressed keratinocytes additionally release IL1, driving dermal endothelial adhesion-molecule expression [33, 34], and IL18 correlates with psoriasis severity and has been identified as a risk factor for atherosclerotic plaque progression [35, 36]. TNF-α and IFN-γ synergism further links endothelial adhesion, barrier integrity, and atherosclerosis [9], with IFN-γ promoting IL1 and IL23 release and driving Th17/Th22 differentiation, and TNF-α, produced broadly by macrophages, T-cell subsets, dendritic cells, adipose tissue, and keratinocytes, directly upregulating CRP [37]. This multi-source production profile is precisely why TNF-α is classified here as a shared effector rather than as an IL23-axis-proximal cytokine [37]. IL17a’s relationship to disease activity remains less consistent across studies [35, 38], though keratinocyte-specific IL17a overexpression in animal models produces circulating inflammatory leukocytosis, elevated blood pressure, and endothelial dysfunction, reversible by neutralizing the downstream marker IL6 [39]. In humans, circulating IL6 and IL17a correlate with disease severity and endothelial inflammation, consistent with but not proof of the same pathway [40].

Taken together, IL23-axis-proximal cytokines act locally and are directly coupled to cutaneous and microvascular pathology, whereas CRP is a systemically integrated signal shaped by adiposity, metabolic status, and multiple cytokine inputs beyond the IL23 axis. No study has yet mapped these two signals onto separate vascular compartments within the same patients over time [15].

## Evidence that skin clearance does not guarantee vascular recovery

Vascular dysfunction in psoriasis spans distinct phenotypes: macrovascular structural remodeling (carotid intima-media thickness), arterial stiffness (pulse wave velocity), and conduit-artery endothelial function (flow-mediated dilation), alongside microvascular phenotypes assessed by laser-Doppler flowmetry and dynamic microvascular imaging [41]. Compared with controls, individuals with psoriasis show higher central and peripheral blood pressure [42], greater carotid intima-media thickness [42–44], and elevated arterial stiffness [45, 46] that tracks cumulative inflammatory burden [46]. Endothelium-independent vasodilation findings are mixed [17, 43, 47, 48], but endothelium-dependent vasodilation is consistently impaired in active disease, at both conduit-artery and cutaneous microvascular levels [43, 47, 48], where NO-mediated vasodilation is inversely correlated with lesional body-surface area [17]. Notably, whatever its lesional concentration, NO in active psoriasis is iNOS-derived and behaves as an inflammatory messenger rather than as a bioavailable vasodilator, so elevated total nitrite/nitrate coexists with reduced NO-dependent vasodilation rather than contradicting it [49]. Serum nitrate/nitrite can be 4-fold higher than in controls, correlates with severity, and normalizes with treatment [22, 50, 51], which suggests, although circulating nitrate/nitrite is also influenced by diet and renal function, that at least part of the vascular signature is linked to cutaneous inflammation rather than being purely metabolic.

Whether treatment resolves this vascular signature is inconsistent. Despite clinically confirmed skin clearance, shared effector cytokines (TNF-α, IFN-γ, IL1β) and the axis-proximal cytokine IL17 can remain elevated across treatment classes, including IL23 blockade, and correlate with BMI and waist circumference even when CRP is not elevated, whereas circulating IL23 itself is not elevated [6]. Conversely, CRP itself can remain elevated after biologic treatment, correlating with metabolic dysfunction, BMI, and visceral adiposity despite optimal skin clearance [5], and this CRP-defined residual inflammatory risk independently predicts adverse cardiovascular events [52]. Female sex predicts residual inflammation despite men typically presenting more severe disease [5, 53], and visceral adipose tissue-derived TNF-α, IL6, and leptin promote Th17 polarization [54], entangling metabolic and psoriatic inflammatory pathways in ways that complicate simple BMI-based stratification. Reported effects of anti-TNF treatment on TNF-α levels [7] and adhesion molecules [33, 34, 55] are similarly inconsistent across studies. Beyond cytokine levels, psoriatic skin retains epigenetically reprogrammed, resident-memory-T-cell-driven inflammatory potential [56, 57], and a residual psoriatic genomic signature associated with endothelial dysfunction persists in clinically remitted skin [58, 59], consistent with relapse rates of 67–91% after treatment discontinuation [2, 60].

Vascular recovery mirrors this inconsistency. IL17a inhibition improves conduit-artery endothelial function without changing arterial stiffness [61]. In psoriatic arthritis, two years of TNF-α inhibition produced no recovery of flow-mediated dilation and slight but significant carotid intima-media progression despite improved disease activity and lipids [7], whereas TNF-α blockade improved vascular function in a psoriasis cohort [62]. Yet other cohorts show preserved endothelial function and arterial stiffness across diverse treatments, independent of skin outcomes [63]. Because nearly all of this evidence comes from post-treatment cross-sections rather than paired pre-post or untreated-comparator designs, it cannot currently distinguish whether these discordant vascular outcomes reflect compartment-specific biology, as we hypothesize, or simply heterogeneous study populations and treatment durations.

## Testable predictions

**Fig. 1:**
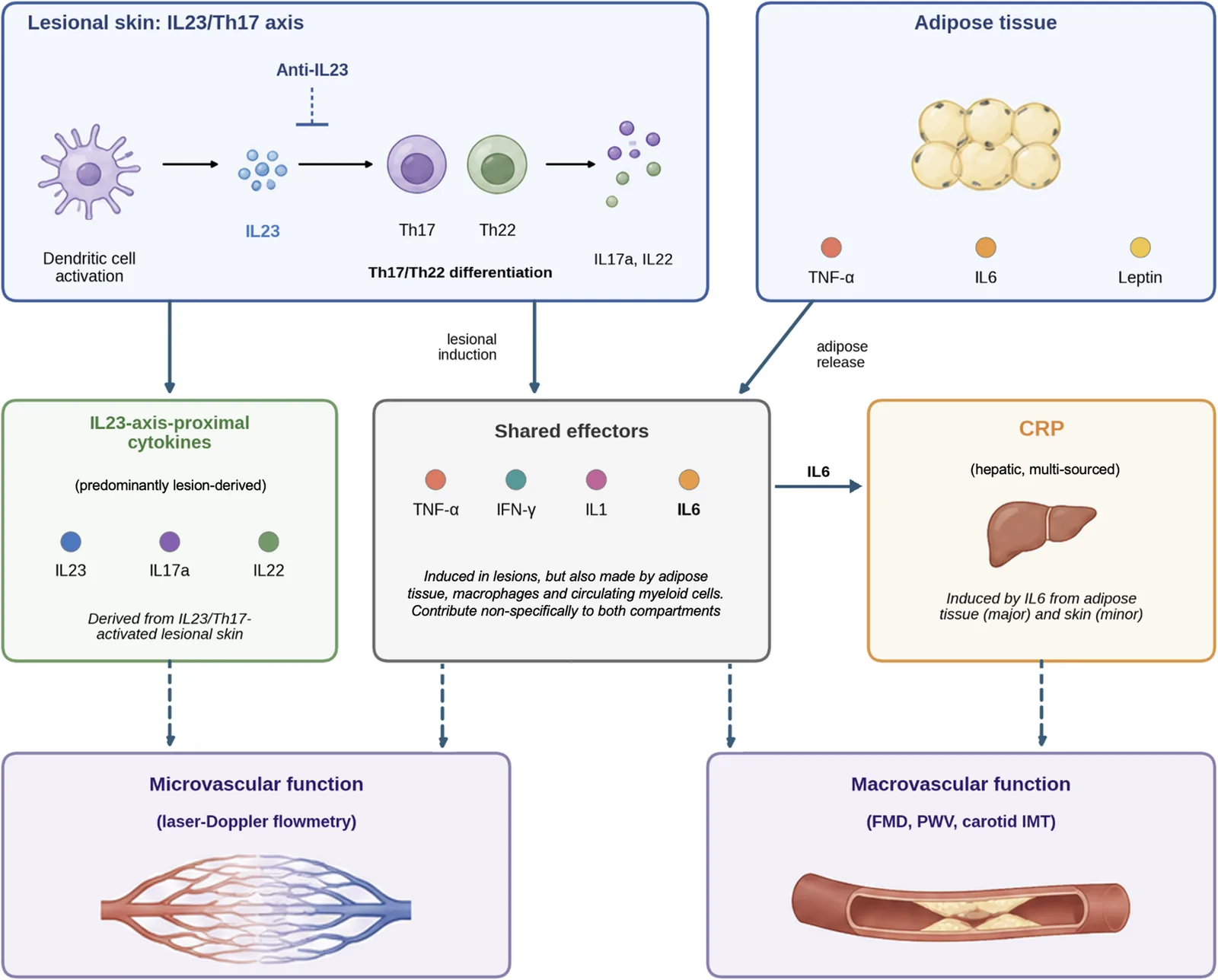
Proposed model of partially dissociable IL23-axis-proximal and C-reactive protein (CRP)-integrated inflammatory signaling and their hypothesized, compartment-specific relationships to microvascular and macrovascular recovery after biologic treatment. IL23-axis-proximal cytokines (IL23, IL17a, IL22) are shown as predominantly lesion-derived. TNF-α, IFN-γ, and IL1 are shown separately as shared effectors, induced within psoriatic lesions but also produced by adipose tissue and circulating myeloid cells, rather than assigned exclusively to either compartment. IL6 is depicted as a shared node receiving both lesional and adipose input and as the dominant hepatic inducer of CRP (solid IL6 arrow), so that the two compartments are coupled, and the hypothesis is one of quantitative rather than absolute dissociation. Dashed lines from IL23-axis-proximal cytokines and from CRP denote the untested compartment-specific links addressed by Predictions 1 and 2; dashed lines from shared effectors denote their non-specific contributions to both vascular compartments

Our hypothesis generates two directly falsifiable predictions (Fig. 1).

- Prediction 1: Microvascular recovery tracks IL23-axis-proximal suppression and tracks CRP only weakly. Cutaneous microvascular endothelial dysfunction scales with lesional disease extent [17], and microvascular abnormalities in the cutaneous and coronary circulations, together with coronary plaque features, relate to disease severity or improve with treatment [17, 64, 65], consistent with, though not yet direct evidence for, dependence of microvascular function on IL23-axis-proximal signaling, whereas CRP and conventional cardiometabolic risk factors such as hypertension and dyslipidemia have not been shown to predict microvascular dysfunction [18, 66]. IL23 blockade with risankizumab produces rapid, substantial skin improvement, with roughly 90% PASI improvement by 16 weeks [67] and significant lesional and epidermal improvement documented as early as 4 weeks [68]. Risankizumab also ranks highly for skin efficacy in meta-analysis, and favorable changes in markers of subclinical atherosclerosis have been reported with IL17/IL23 inhibitors in observational data [69, 70]. If our model is correct, laser-Doppler-measured cutaneous microvascular function should track axis-proximal suppression closely and track ΔCRP only weakly. We state this in testable quantitative form: the partial correlation between Δmicrovascular function and ΔIL17a adjusted for ΔCRP should remain significant, the partial correlation with ΔCRP adjusted for ΔIL17a should be negligible, demonstrated by an equivalence test (90% confidence interval lying entirely within ± 0.3) rather than by non-significance alone, and the zero-order correlation with ΔIL17a should exceed that with ΔCRP, with the difference statistically significant on a test for dependent correlations; the design below is powered to detect a difference of 0.3. This is the falsifiable content of the claimed dissociation in Prediction 1; strict statistical independence is neither claimed nor expected, given the shared IL6 node. Such a dissociation is plausible, since systemic inflammation and atherosclerotic risk are not reliably ameliorated alongside cutaneous improvement in the same patients [5].

- Prediction 2: Persistent CRP elevation despite skin clearance flags blunted macrovascular recovery, even when IL23-axis-proximal signaling is suppressed. Recent cohort data show that shared effector cytokines (TNF-α, IFN-γ, IL1β) and the axis-proximal cytokine IL17 can remain elevated even when CRP is not elevated [6], while CRP itself can remain elevated in patients with metabolic dysfunction, higher BMI, and visceral adiposity despite optimal skin-directed therapy [5]. If CRP is the operative macrovascular signal, then patients with persistent CRP elevation, even when axis-proximal signaling is suppressed, should show blunted recovery in arterial stiffness and conduit-artery endothelial function, consistent with reports that improved psoriatic arthritis activity can coexist with persistent endothelial dysfunction and progression of carotid atherosclerosis [7], even though arterial function is preserved in some well-controlled cohorts [63].

Both predictions can be tested using existing prospective cohorts [5, 6] reanalyzed with paired micro- and macrovascular imaging, or through a purpose-built longitudinal design: patients initiating IL23 blockade undergo serial measurement of IL23-axis-proximal cytokines (IL23, IL17a, IL22, by high-sensitivity immunoassay given their low circulating concentrations), shared effectors including IL6, high-sensitivity CRP, cutaneous microvascular function (laser-Doppler flowmetry), and macrovascular function (flow-mediated dilation, pulse wave velocity, carotid intima-media thickness) at baseline and at 4, 16, and 52 weeks. The primary analysis is a double dissociation tested with partial correlations and cross-lagged models: microvascular recovery correlating with axis-proximal suppression after adjustment for ΔCRP, and macrovascular recovery correlating with ΔCRP after adjustment for axis-proximal cytokines, with adiposity (visceral fat or waist circumference) entered as a covariate so that the adipose contribution to IL6 and CRP is modeled rather than assumed away. Because IL6 is the shared node, we additionally prespecify a mediation analysis partitioning ΔCRP into lesion-attributable and adiposity-attributable components. Approximately 100 patients would provide roughly 80% power to detect a difference of 0.3 between dependent correlations (for example, 0.5 versus 0.2, assuming a correlation of 0.3 between ΔIL17a and ΔCRP; two-sided α = 0.05; Steiger’s test for dependent correlations); allowing for approximately 15% attrition over 52 weeks, about 120 patients should be enrolled, and larger samples would be needed for smaller differences. At this sample size, the equivalence test for the ΔCRP partial correlation has approximately 80% power with a bound of ± 0.3 if the true partial correlation is near zero; a tighter bound of ± 0.2 would require roughly 200 evaluable patients. The mediation analysis should be considered exploratory at this sample size. Support for the hypothesis requires that each compartment be predicted by its assigned biomarker after adjustment for the other. Comparable prediction of both compartments by either biomarker would refute it.

## Clinical implications and future directions

If confirmed, this compartmentalized model could carry direct clinical implications. Skin clearance alone would no longer be a sufficient proxy for cardiovascular risk reduction. Instead, clinicians could use IL23-axis-proximal cytokine trajectory and CRP as complementary, mechanism-specific biomarkers, one flagging residual microvascular risk, the other residual macrovascular and cardiometabolic risk, to guide adjunctive management (e.g., weight and metabolic intervention for persistent CRP elevation, or closer monitoring of microvascular complications for persistent IL23-axis-proximal activity) even in patients with excellent cutaneous response.

Longitudinal designs of the kind proposed above would also clarify the role of relapse biology. Because psoriasis relapse may be driven in part by persistent tissue-resident memory T cells that retain inflammatory potential in clinically resolved skin [56, 58], tracking systemic inflammation and both vascular compartments through and beyond clinical remission could identify early biomarkers of relapse, distinguish durable remission from subclinical disease activity, and inform preventive strategies that address dermatologic and cardiovascular outcomes together, rather than treating skin clearance as a sufficient endpoint in itself.

## Limitations of the hypothesis

Several limitations constrain the hypothesis as stated. First, the supporting evidence is largely cross-sectional or drawn from post-treatment observations, and the quantitative bounds placed on the shared IL6 node come from overweight to severely obese, non-psoriatic populations [30, 31] and from patients treated with TNF-α and IL12/23 antagonists rather than IL23-selective agents [32]. Second, in well-controlled psoriasis treated with topical, conventional, or biologic therapy, circulating IL6 has recently been shown to be predicted mainly by residual circulating cytokines (IL12p70, IFN-γ, IL1β, IL17) rather than by glycemic, hepatic, or overall visceral-adiposity indices, although IL6 rose non-linearly across a visceral adiposity index range of approximately 1.3–2.2 [71]. If this pattern extends to CRP, the psoriasis-related share of the IL6–CRP route may be larger than estimated here, and Prediction 1 would be correspondingly harder to satisfy. Third, circulating IL23-axis-proximal cytokines are present at low concentrations and are imperfect surrogates for lesional activity: circulating IL23 is not elevated in successfully treated patients even when IL17 remains so [6], and near-uniform suppression of IL17a under IL23 blockade may restrict the variance available for correlation-based tests, so lesional transcriptomic measures may be needed alongside serum assays. Circulating IL17a is also not exclusively lesional: IL17-producing cells expand in obesity [54], and residual circulating IL17 tracks BMI and waist circumference in treated patients [6], so a non-cutaneous fraction of circulating IL17a would attenuate the contrast tested in Prediction 1. Fourth, the model assumes that cutaneous microvascular function measured by laser-Doppler flowmetry is representative of microvascular beds elsewhere, which has not been established, and both micro- and macrovascular measures carry appreciable measurement variability. Finally, the model is restricted to the IL23/IL17 and IL6/CRP routes; other contributors to vascular risk, including lipids, blood pressure, and psoriatic arthritis, are treated as covariates rather than modeled mechanistically.

## Data Availability

Not applicable. No new data were generated or analyzed for this manuscript. All findings discussed derive from previously published, cited studies.
